# Supplementary material for: Short-term effects of hurricanes Maria and Irma on forest birds of Puerto Rico
Source: PLoS One. 2019 Jun 11;14(6):e0214432. doi: 10.1371/journal.pone.0214432 (PMC6559628; doi:10.1371/journal.pone.0214432)
Supplement: S1 Appendix — (DOCX) [file pone.0214432.s001.docx]

sink("PRModelPhiDetCovReducedSet2.txt")

cat("

model{

## Prior for the community level hyper parameter on mean occupancy

mean.mu.u ~ dunif(0,1)

mean.u <- log(mean.mu.u) - log(1-mean.mu.u) # alternative formation of the logit

## Prior for the community level hyper parameter on mean detection

mean.mu.v ~ dunif(0,1)

mean.v <- log(mean.mu.v) - log(1-mean.mu.v)

## Prior for the community level hyper parameter for precision of occupancy and detection

sd.mu.u ~ dunif(0,10)

#tau.mu.u ~ pow(sd.mu.u,-2)

tau.mu.u <- 1/(sd.mu.u*sd.mu.u)

sd.mu.v ~ dunif(0,10)

#tau.mu.v ~ pow(sd.mu.v, -2)

tau.mu.v <- 1/(sd.mu.v*sd.mu.v)

## Prior for regression coefficients for effects of time and date on detectability

mu.betaDate ~ dnorm(0, 0.1)

sd.betaDate ~ dunif(0, 1)

tau.betaDate <- 1/(sd.betaDate*sd.betaDate)

mu.betaTime ~ dnorm(0, 0.1) #prior for the survey-time coefficient

sd.betaTime ~ dunif(0, 1)

tau.betaTime <- 1/(sd.betaTime*sd.betaTime)

mu.betaPrevDet ~ dnorm(0, 0.1)

sd.betaPrevDet ~ dunif(0, 1)

tau.betaPrevDet <- 1/(sd.betaPrevDet*sd.betaPrevDet)

### Loop over all species i

for (i in 1:nR2) {

#### Create priors for species i from the community level prior distributions

##### Prior distribution for intercept term of species occupancy

mu.u[i] ~ dnorm(mean.u, tau.mu.u)

sd.u[i] ~ dunif(0,10)

#tau.u[i] <- pow(sd.u[i], -2)

tau.u[i] <- 1/(sd.u[i]*sd.u[i])

u[i] ~ dnorm(mu.u[1], tau.u[i])

##### Prior distribution for intercept term of species detection

mu.v[i] ~ dnorm(mean.v, tau.mu.v)

sd.v[i] ~ dunif(0,10)

#tau.v[i] <- pow(sd.v[i], -2)

tau.v[i] <- 1/(sd.v[i]*sd.v[i])

v[i] ~ dnorm(mu.v[i], tau.v[i])

##### Prior for phi and gamma

phi[i] ~ dunif(0,1)

gam[i] ~ dunif(0,1)

##### Prior for detection covariates

betaDate[i] ~ dnorm(mu.betaDate, tau.betaDate)

betaTime[i] ~ dnorm(mu.betaTime, tau.betaTime)

betaPrevDet[i] ~ dnorm(mu.betaPrevDet, tau.betaPrevDet)

### Create a loop to estimate the Z matrix (true occurrence for species i

### at point j) for all J sites.

##### Occurence, year 1

for (j in 1:J) {

for(y in 1:(Y-1)) {

logit(psi[j,y,i]) <- u[i]

Z[j,y,i] ~ dbern(psi[j,y,i])

} #year 1

##### Occurence in subsequent years

for(y in 2:Y) {

##### occurrence in year 2

psi[j,y,i] <- (Z[j,y-1,i] * phi[i] + (1 - Z[j,y-1,i]) * gam[i])

Z[j,y,i] ~ dbern(psi[j,y,i])

} # Year 2

##### Detection

for (y in 1:Y) {

for (k in 1:4) { #K[j]

logit(p[j,y,k,i]) <- v[i] + betaDate[i]*date.array[j,y,k] + betaTime[i]*time.array[j,y,k] + betaPrevDet[i]*detections[j,y,k,i]

mu.y[j,y,k,i] <- p[j,y,k,i]*Z[j,y,i]

X[j,y,k,i] ~ dbern(mu.y[j,y,k,i])

##### Observed deviance

dev[j,y,k,i] <- X[j,y,k,i]*log(mu.y[j,y,k,i]) + (1-X[j,y,k,i])*log(1-mu.y[j,y,k,i])

###### Predict new observation and compute deviance

y.new[j,y,k,i] ~ dbern(mu.y[j,y,k,i])

dev.sim[j,y,k,i] <- y.new[j,y,k,i]*log(mu.y[j,y,k,i]) + (1-y.new[j,y,k,i])*log(1-mu.y[j,y,k,i])

} #rep

} # year detections

} # site

} #species

### Derived values

sum.dev <- sum(dev[,,,])

sum.dev.sim <- sum(dev.sim[,,,])

test <- step(sum.dev.sim - sum.dev)

bpvalue <- mean(test)

for (i in 1:nR2) {

deltaZ[i] <- (sum(Z[,2,i]) - sum(Z[,1,i]))

deltaOcc[i] <- (sum(Z[,2,i]))/(sum(Z[,1,i]))

deltaV[i] <- v[i,2] - v[i,1]

}

#Finish writing the text file into a document

} #model

",fill = TRUE)

sink()
