# Supplementary figures and images for: Short-term effects of hurricanes Maria and Irma on forest birds of Puerto Rico
Source: PLoS One. 2019 Jun 11;14(6):e0214432. doi: 10.1371/journal.pone.0214432 (PMC6559628; doi:10.1371/journal.pone.0214432)

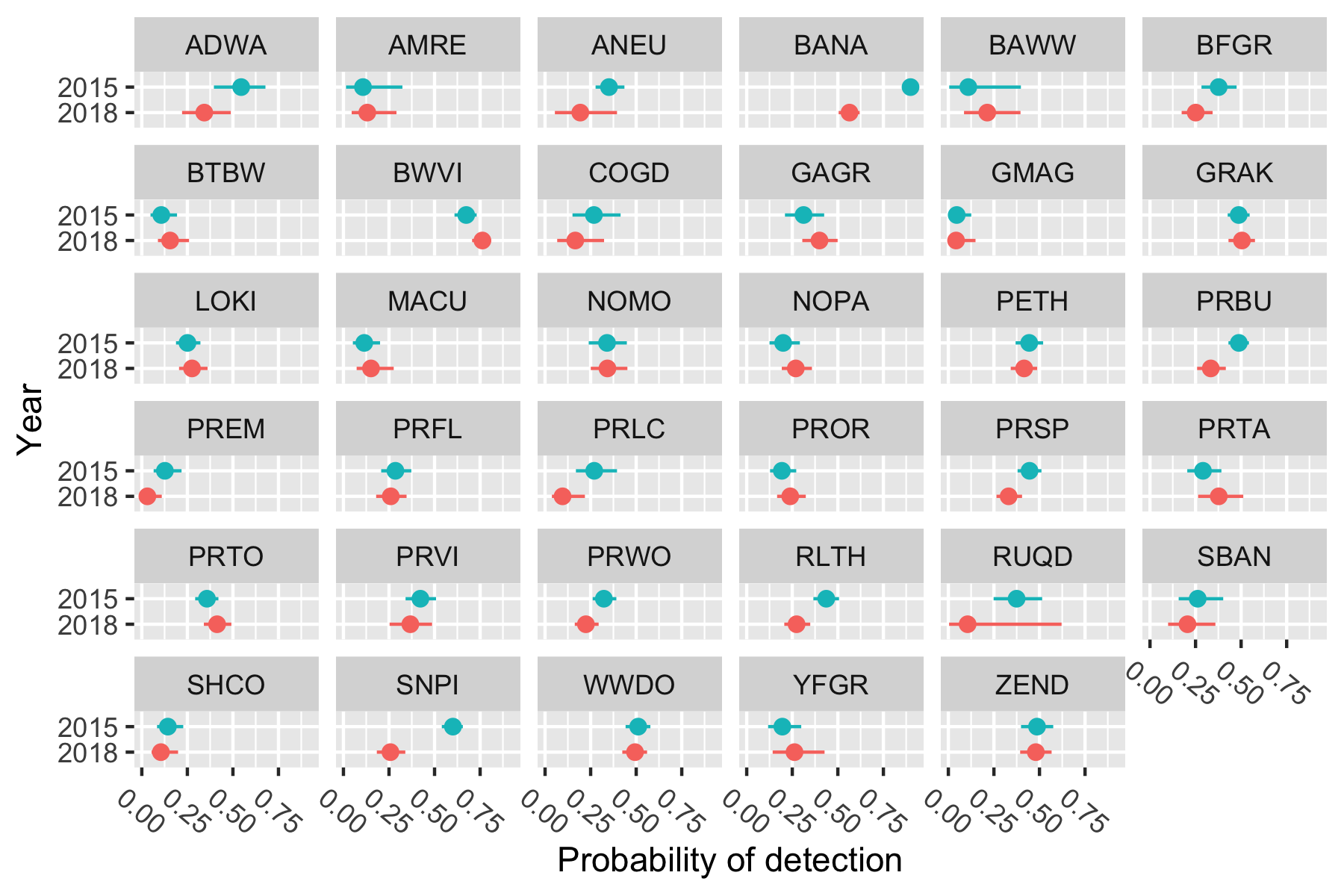

Supplement: S1 Fig — Dots show the mean of the posterior samples, and lines represent the 95% credible interval. For more information on species codes and names, see S1 Table. (TIF) [file pone.0214432.s002.tif]

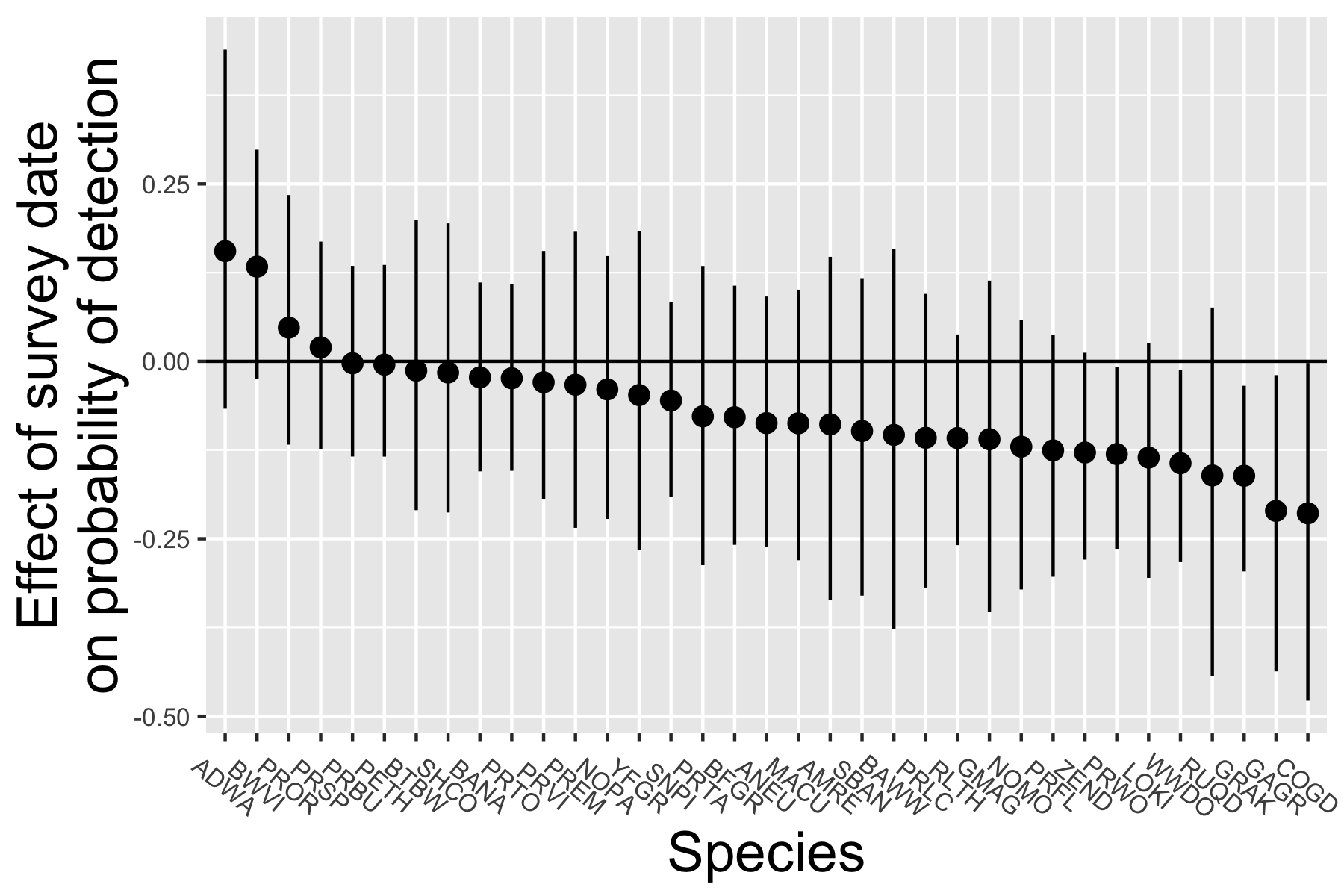

Supplement: S2 Fig — Dots show the mean of the posterior samples, and lines represent the 95% credible interval. The solid horizontal line represents no effect of date on probability of detection; species falling below the line had lower detectability during surveys conducted later in the season, whereas species above the line had higher detectability during surveys conducted later in the season. For more information on species codes and names, see S1 Table. (TIF) [file pone.0214432.s003.tif]

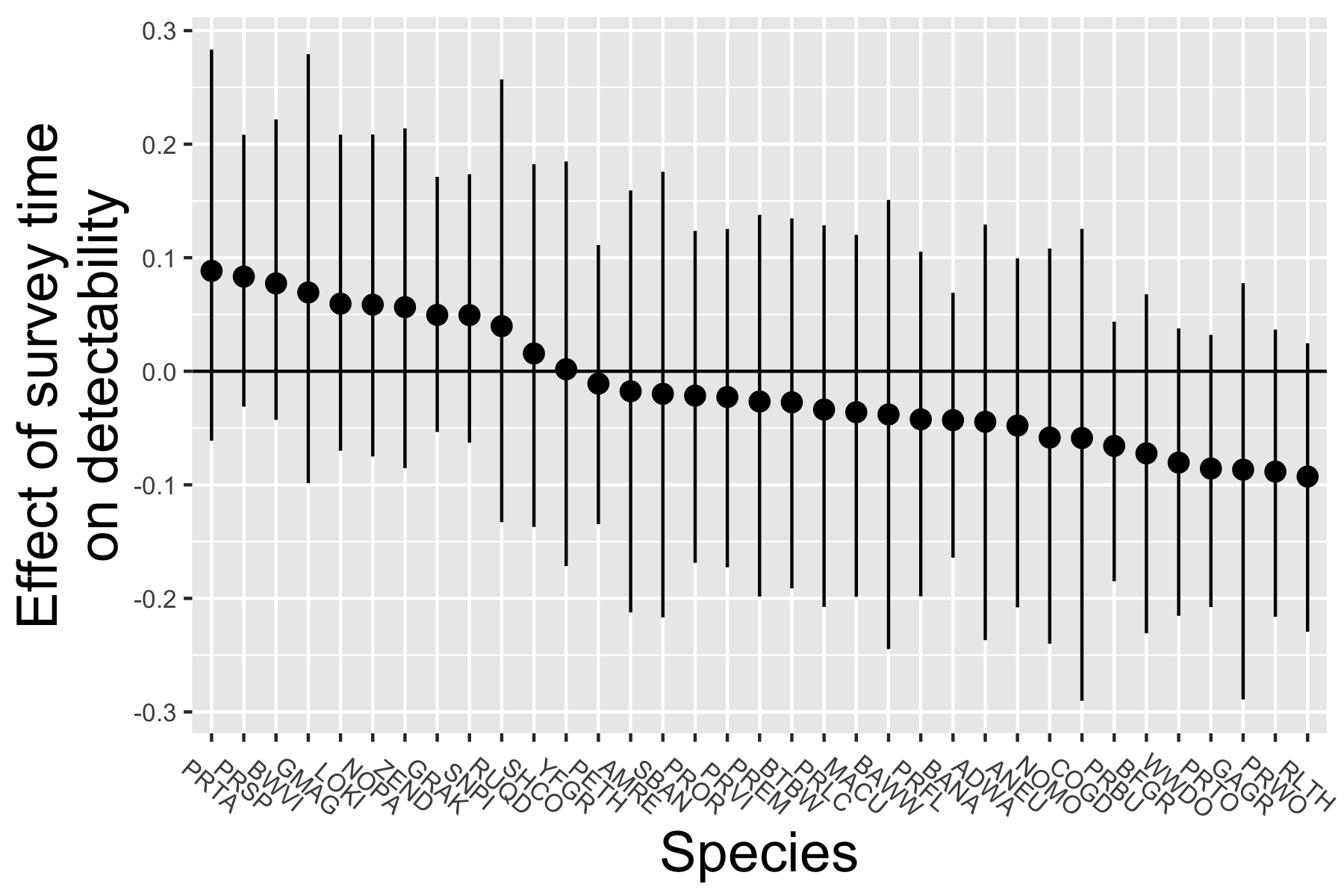

Supplement: S3 Fig — Dots show the mean of the posterior samples, and lines represent the 95% credible interval. The solid horizontal line represents no effect of time on probability of detection; species falling below the line had lower detectability during surveys conducted later in the day, whereas species above the line had higher detectability during surveys conducted later in the day. For more information on species codes and names, see S1 Table. (TIF) [file pone.0214432.s004.tif]

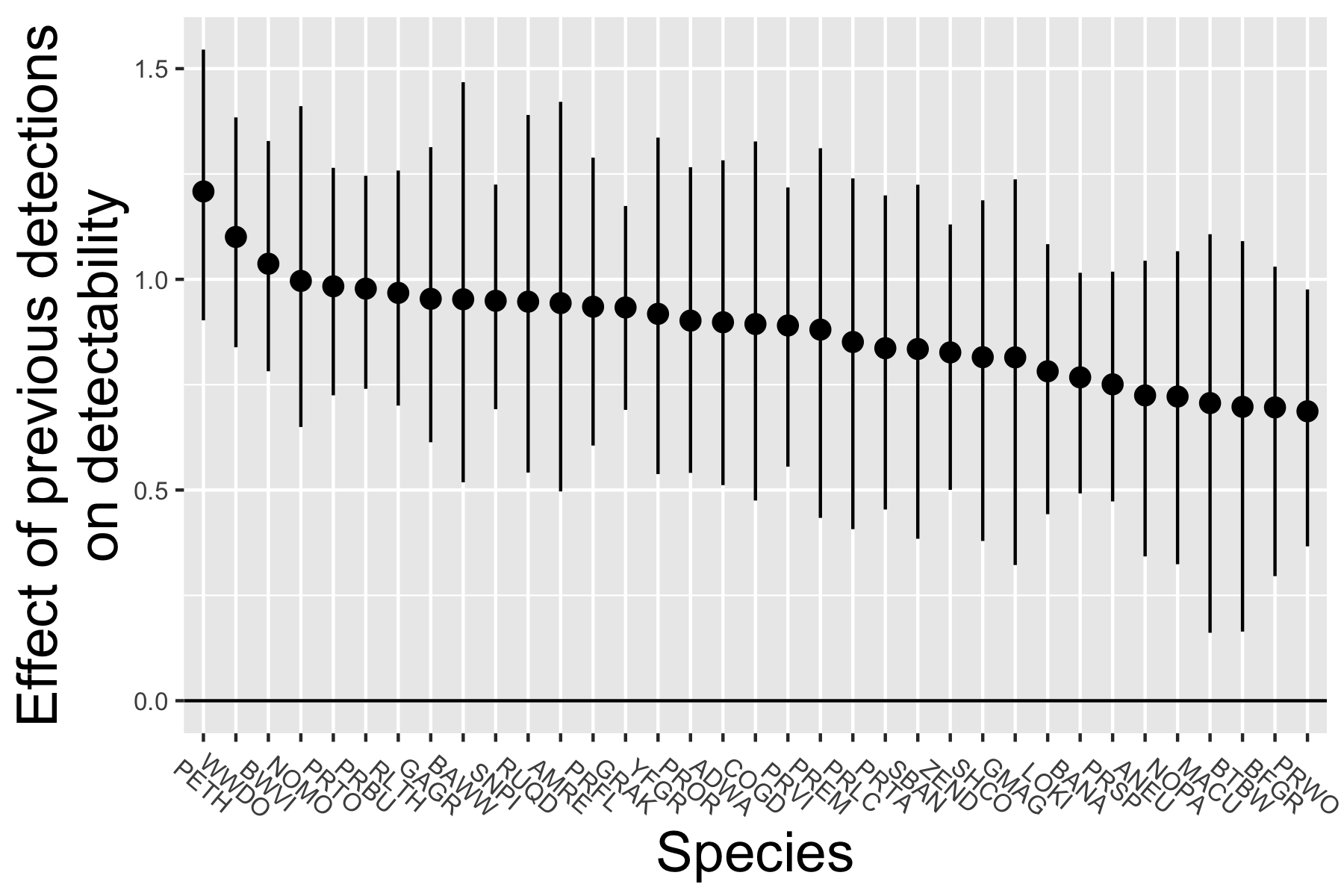

Supplement: S4 Fig — Dots show the mean of the posterior samples, and lines represent the 95% credible interval. For species with estimated values >0, the probability that an observer detected the species increased if they had also detected it during previous surveys. For more information on species codes and names, see S1 Table. (TIF) [file pone.0214432.s005.tif]

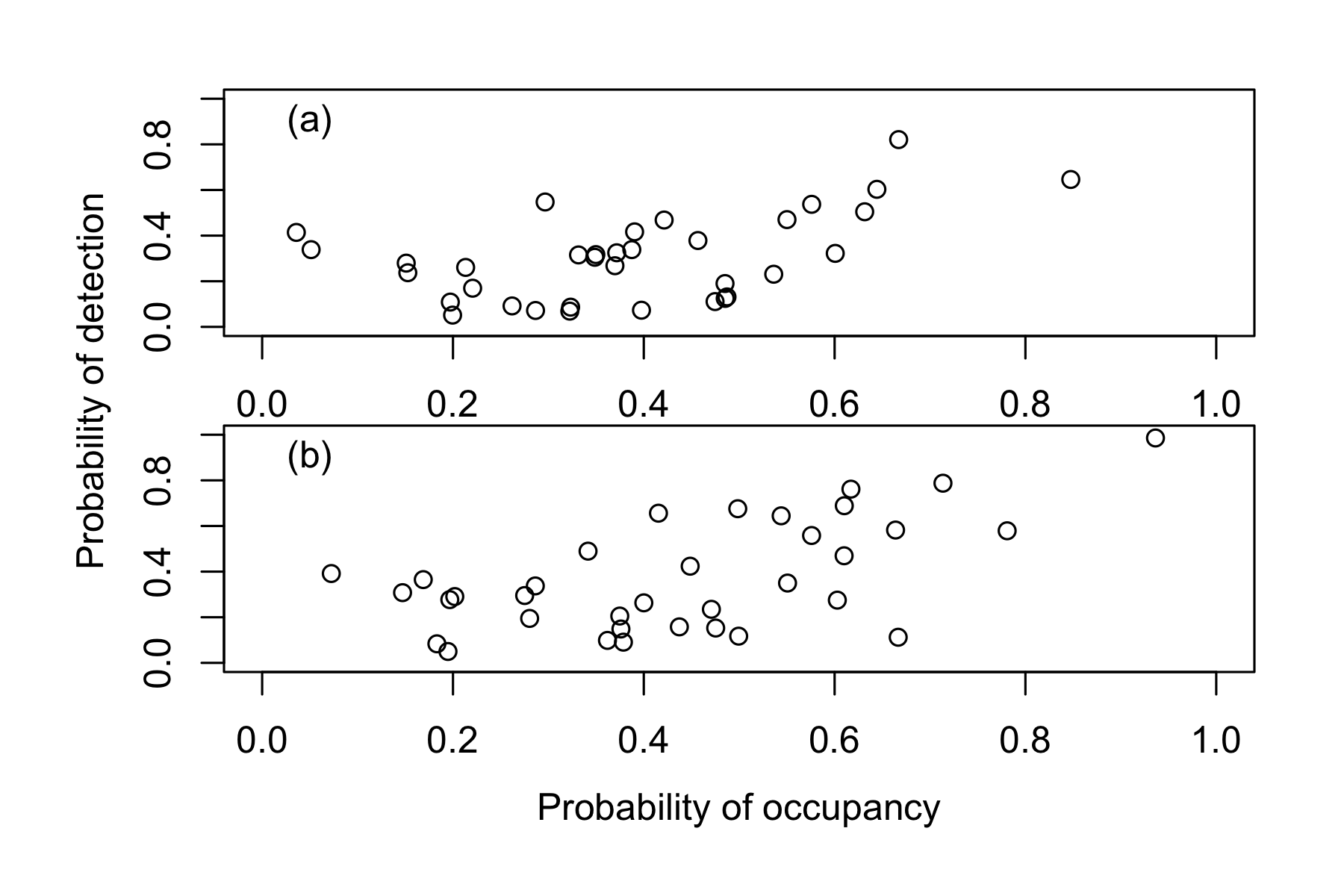

Supplement: S5 Fig — Relationships between the probability of detection and the probability of site occupancy among 35 species of forest birds on Puerto Rico surveyed during a winter before (2015; a) and after (2018; b) passage of hurricanes Irma and Maria. (TIF) [file pone.0214432.s006.tif]

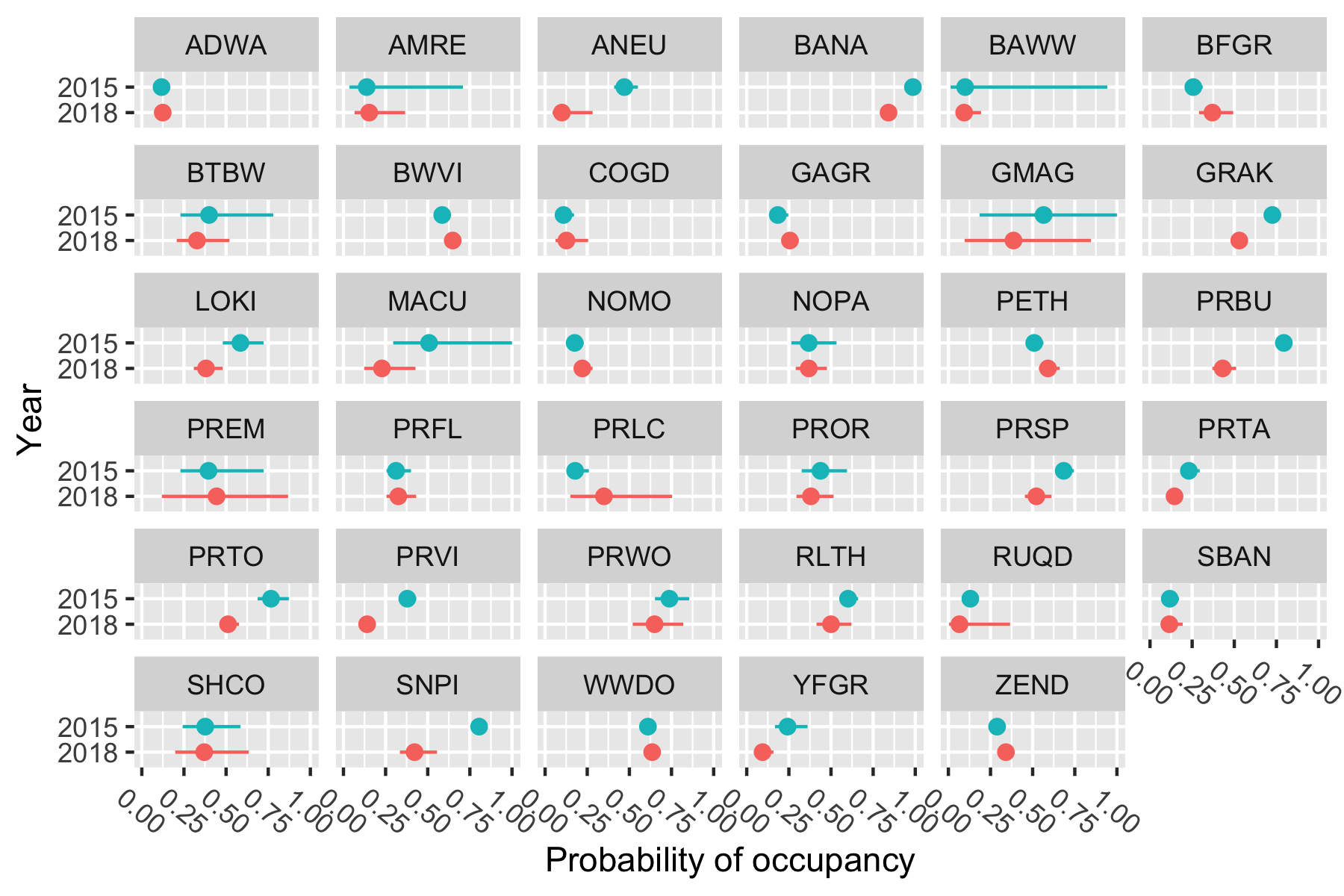

Supplement: S6 Fig — Dots show the mean of the posterior samples, and lines represent the 95% credible interval. For more information on species codes and names, see S1 Table. (TIF) [file pone.0214432.s007.tif]
