# Supplementary material for: Short-term effects of hurricanes Maria and Irma on forest birds of Puerto Rico
Source: PLoS One. 2019 Jun 11;14(6):e0214432. doi: 10.1371/journal.pone.0214432 (PMC6559628; doi:10.1371/journal.pone.0214432)
Supplement: S1 Table — Species in bold were included in the formal analysis of occupancy; English common names and 4-letter codes are provided as well for these species. (DOCX) [file pone.0214432.s008.docx]

**S1 Table. Percentage of sites at which each species was detected and total number of sites at which a species was detected during surveys conducted at 227 points (186 surveyed in both years, 25 surveyed only in 2015, and 16 surveyed only in 2018) in forested areas across Puerto Rico during January - March 2015 and 2018.** Species in bold were included in the formal analysis of occupancy; English common names and 4-letter codes are provided as well for these species.

|  | Sites detected (%) | | Sites detected (N) | |  |
| --- | --- | --- | --- | --- | --- |
| Species | 2015 | 2018 | 2015 | 2018 | Dietary guild^1^ |
| ***Coereba flaveola***  **(Bananaquit, BANA)** | 98.6 | 81.2 | 208 | 164 | N |
| ***Patagioenas squamosa***  **(Scaly-naped Pigeon, SNPI)** | 78.2 | 28.7 | 165 | 58 | F |
| ***Vireo altiloquus***  **(Black-whiskered Vireo, BWVI)** | 57.8 | 65.3 | 122 | 132 | I |
| ***Tyrannus dominicensis***  **(Gray Kingbird, GRAK)** | 67.3 | 49.5 | 142 | 100 | I |
| ***Zenaida asiatica***  **(Zenaida Dove, ZEND)** | 57.3 | 59.4 | 121 | 120 | G |
| ***Loxigilla portoricensis***  **(Puerto Rican Bullfinch, PRBU)** | 74.4 | 33.7 | 157 | 68 | F |
| ***Todus mexicanus***  **(Puerto Rican Tody, PRTO)** | 63.0 | 45.0 | 133 | 91 | I |
| ***Spindalis portoricensis***  **(Puerto Rican Spindalis, PRSP)** | 61.6 | 41.6 | 130 | 84 | F |
| ***Melanerpes portoricensis***  **(Puerto Rican Woodpecker, PRWO)** | 57.3 | 40.6 | 121 | 82 | I |
| ***Margarops fuscatus***  **(Pearly-eyed Thrasher, PETH)** | 46.0 | 52.0 | 97 | 105 | O |
| ***Turdus plumbeus***  **(Red-legged Thrush, RLTH)** | 53.6 | 35.1 | 113 | 71 | O |
| ***Tyrannus caudifasciatus***  **(Loggerhead Kingbird, LOKI)** | 38.9 | 26.7 | 82 | 54 | I |
| ***Zenaida aurita***  **(White-winged Dove, WWDO)** | 27.5 | 31.7 | 58 | 64 | G |
| ***Icterus portoricensis***  **(Puerto Rican Oriole, PROR)** | 24.6 | 24.8 | 52 | 50 | O |
| ***Setophaga americana***  **(Northern Parula, NOPA)** | 20.9 | 25.7 | 33 | 52 | I |
| ***Tiaris bicolor***  **(Black-faced Grassquit, BFGR)** | 21.3 | 24.8 | 45 | 50 | G |
| ***Myiarchus antillarum***  **(Puerto Rican Flycatcher, PRFL)** | 22.7 | 22.3 | 48 | 45 | I |
| ***Vireo latimeri***  **(Puerto Rican Vireo, PRVI)** | 33.6 | 10.9 | 71 | 22 | I |
| ***Euphonia musica***  **(Antillean Euphonia, ANEU)** | 38.4 | 4.5 | 81 | 9 | F |
| ***Quiscalus niger***  **(Greater Antillean Grackle, GAGR)** | 13.7 | 21.8 | 29 | 44 | O |
| ***Molothrus bonariensis***  **(Shiny Cowbird, SHCO)** | 16.6 | 12.4 | 35 | 25 | G |
| ***Mimus polyglottos***  **(Northern Mockingbird, NOMO)** | 13.7 | 17.3 | 29 | 35 | O |
| ***Setophaga caerulescens***  **(Black-throated Blue Warbler, BTBW)** | 13.7 | 15.3 | 29 | 31 | I |
| ***Coccyzus minor***  **(Mangrove Cuckoo, MACU)** | 18.5 | 9.9 | 39 | 20 | O |
| ***Nesospingus speculiferus***  **(Puerto Rican Tanager, PRTA)** | 16.1 | 11.4 | 34 | 23 | F |
| ***Coccyzus vieilloti***  **(Puerto Rican Lizard-Cuckoo, PRLC)** | 12.3 | 9.9 | 26 | 20 | O |
| ***Chlorostilbon maugaeus***  **(Puerto Rican Emerald, PREM)** | 15.6 | 4.5 | 33 | 9 | N |
| ***Tiaris olivaceus***  **(Yellow-faced Grassquit, YFGR)** | 13.7 | 5.9 | 29 | 12 | G |
| *Buteo jamaicensis* | 1.9 | 3.0 | 4 | 6 | C |
| ***Setophaga adelaidae***  **(Adelaide’s Warbler, ADWA)** | 10.0 | 8.4 | 21 | 17 | I |
| ***Anthracothorax viridis***  **(Green Mango, GRMA)** | 8.0 | 6.0 | 18 | 10 | N |
| ***Crotophaga ani***  **(Smooth-billed Ani, SBAN)** | 8.1 | 5.9 | 17 | 12 | O |
| ***Columbina passerina***  **(Common Ground Dove, COGD)** | 7.1 | 5.5 | 15 | 11 | G |
| ***Geotrygon montana***  **(Ruddy Quail-Dove, RUQD)** | 10.9 | 0.5 | 23 | 1 | F |
| *Megascops nudipes* | 8.5 | 2.0 | 18 | 4 | C |
| *Falco sparverius* | 1.4 | 7.4 | 3 | 15 | C |
| ***Setophaga ruticilla***  **(American Redstart, AMRE)** | 3.3 | 5.4 | 7 | 11 | I |
| *Bubulcus ibis* | 1.4 | 2.5 | 5 | 4 | O |
| *Estrilda melpoda* | 7.1 | 0.5 | 15 | 1 | G |
| *Passer domesticus* | 4.7 | 1.5 | 10 | 3 | O |
| ***Mniotilta varia***  **(Black-and-white Warbler, BAWW)** | 1.4 | 5.0 | 3 | 10 | I |
| *Cathartes aura* | 1.9 | 4.5 | 4 | 9 | C |
| *Progne dominicensis* | 1.4 | 3.5 | 3 | 7 | I |
| *Ardea alba* | 0.5 | 5.0 | 1 | 10 | C |
| *Catharus bicknelli* | 0.5 | 5.0 | 1 | 10 | I |
| *Icterus icterus* | 3.8 | 1.5 | 8 | 3 | O |
| *Petrochelidon fulva* | 0.5 | 5.0 | 1 | 10 | I |
| *Columba livia* | 0.5 | 4.5 | 1 | 9 | G |
| *Contopus latirostris* | 3.3 | 1.5 | 7 | 3 | I |
| *Elaenia martinica* | 2.4 | 2.0 | 5 | 4 | F |
| *Brotogeris versicolurus* | 1.9 | 2.0 | 4 | 4 | F |
| *Setophaga angelae* | 3.3 | 0.5 | 7 | 1 | I |
| *Parkesia motacilla* | 2.4 | 1.0 | 5 | 2 | I |
| *Setophaga discolor* | 1.4 | 2.0 | 3 | 4 | I |
| *Anthracothorax dominicus* | 1.9 | 1.0 | 4 | 2 | N |
| *Geotrygon chrysia* | 2.4 | 0.5 | 5 | 1 | F |
| *Butorides virescens* | 0.5 | 2.0 | 1 | 4 | C |
| *Spermestes cucullata* | 1.9 | 0.5 | 4 | 1 | G |
| *Setophaga coronata* | 0 | 2.0 | 0 | 4 | I |
| *Patagioenas leucocephala* | 0.5 | 1.0 | 1 | 2 | F |
| *Setophaga tigrina* | 0.9 | 0.5 | 2 | 1 | N |
| *Setophaga virens* | 0.9 | 0.5 | 2 | 1 | I |
| *Accipiter striatus* | 0.5 | 0.5 | 1 | 1 | C |
| *Seiurus aurocapilla* | 0.5 | 0.5 | 1 | 1 | I |
| *Setophaga citrina* | 0.9 | 0 | 2 | 0 | I |
| *Anas discors* | 0 | 1.0 | 0 | 2 | O |
| *Egretta thula* | 0 | 1.0 | 0 | 2 | C |
| *Geothlypis formosa* | 0 | 1.0 | 0 | 2 | I |
| *Cypseloides niger* | 0.5 | 0 | 1 | 0 | I |
| *Eulampis holosericeus* | 0.5 | 0 | 1 | 0 | N |
| *Eupsittula canicularis* | 0.5 | 0 | 1 | 0 | F |
| *Orthorhyncus cristatus* | 0.5 | 0 | 1 | 0 | N |
| *Setophaga magnolia* | 0.5 | 0 | 1 | 0 | I |
| *Thalasseus maximus* | 0.5 | 0 | 1 | 0 | C |
| *Ardea herodias* | 0 | 0.5 | 0 | 1 | C |
| *Buteo platypterus* | 0 | 0.5 | 0 | 1 | C |
| *Charadrius vociferus* | 0 | 0.5 | 0 | 1 | I |
| *Egretta caerulea* | 0 | 0.5 | 0 | 1 | C |
| *Gallinula galeata* | 0 | 0.5 | 0 | 1 | O |
| *Himantopus mexicanus* | 0 | 0.5 | 0 | 1 | I |
| *Myiopsitta monachus* | 0 | 0.5 | 0 | 1 | F |
| *Nyctanassa violacea* | 0 | 0.5 | 0 | 1 | C |
| *Pandion haliaetus* | 0 | 0.5 | 0 | 1 | C |
| *Passerina caerulea* | 0 | 0.5 | 0 | 1 | G |
| *Podilymbus podiceps* | 0 | 0.5 | 0 | 1 | O |

^1^Nectarivore (N),Frugivore (F), Insectivore (I), Granivore (G), Omnivore (O), Carnivore(C)
